# Anatomy of inferior end of palatopharyngeus: its contribution to upper esophageal sphincter opening

**DOI:** 10.1007/s00405-020-06437-2

**Published:** 2020-10-20

**Authors:** Keiko Fukino, Masahiro Tsutsumi, Akimoto Nimura, Koh Miwa, Takashi Ono, Keiichi Akita

**Affiliations:** 1 Department of Orthodontic Science, Graduate School of Medical and Dental Sciences, Tokyo Medical and Dental University (TMDU), 1-5-45 Yushima, Bunkyo-ku, Tokyo, 113-8549 Japan; 2 Department of Clinical Anatomy, Graduate School of Medical and Dental Sciences, Tokyo Medical and Dental University (TMDU), 1-5-45 Yushima, Bunkyo-ku, Tokyo, 113-8519 Japan; 3 Department of Functional Joint Anatomy, Graduate School of Medical and Dental Science, Tokyo Medical and Dental University, Tokyo, Japan

**Keywords:** Palatopharyngeus, Inferior constrictor, Upper esophageal sphincter, Swallowing, Deglutition disorder

## Abstract

**Purpose:**

The palatopharyngeus is one of the longitudinal pharyngeal muscles which contributes to swallowing. It is reported that the palatopharyngeus has muscle bundles in various directions and with attachment sites, and each muscle bundle has a specific function. Although previous reports suggest that the palatopharyngeus is partly interlaced with some parts of the inferior constrictor, the precise relationship remains unclear. The purpose of this study was to examine the precise manner of the connection between the palatopharyngeus and inferior constrictor, and to examine the histological characteristics of this connection.

**Methods:**

We examined 15 halves of nine heads from Japanese cadavers (average age: 76.1 years); 12 halves, macroscopically, and three halves, histologically.

**Results:**

Our observation suggests that the palatopharyngeus spreads radially on the inner aspect of the pharyngeal wall. The most inferior portion of the palatopharyngeus extended to the inner surface of the cricopharyngeal part of the inferior constrictor. Histological analysis showed that the inferior end of the palatopharyngeus continued into the dense connective tissue located at the level of the cricoid cartilage. The dense connective tissue not only covered the inner surface of the inferior constrictor but also entered its muscle bundles and enveloped them.

**Conclusion:**

Therefore, the palatopharyngeus interlaced the cricopharyngeal part of the inferior constrictor through the dense connective tissues. The findings of this study show that the palatopharyngeus may act on the upper esophageal sphincter directly and help in its opening with the aid of the pulling forces in the superolateral direction.

## Introduction

The opening of the upper esophageal sphincter (UES), which is located on the cricopharyngeal part of the inferior constrictor and the uppermost part of the esophagus [1–4], is one of the essential events required for proper swallowing [5, 6]. In general, the UES opening occurs as a result of the elevation of the hyolaryngeal complex and the relaxation of the cricopharyngeal part of the inferior constrictor [2, 7–11]. Therefore, the mechanism of the UES opening has been considered a passive event, and a few studies have focused on whether any anatomical structures directly act on the UES opening. The evaluation of mechanisms used by these anatomical structures may contribute to the treatment and rehabilitation of dysphagia.

The palatopharyngeus is one of the essential muscles required in proper swallowing, because it contributes to its various events, such as the movement of the soft palate, the shortening of the pharynx, and the elevation of the hyolaryngeal complex [3, 7, 8, 12–14]. The evidence of these contributions has been anatomically explained based on its attachments to the soft palate, the pharyngeal raphe, the epiglottis, and the thyroid cartilage [15–20]. Fukino et al*.* [20] reported that the palatopharyngeus spreads radially and extends inferiorly. However, the precise manner of the connection between the palatopharyngeus and the inferior constrictor has rarely been discussed [17], and its histological characteristics remain unclear. Thus, understanding the precise characteristics of the connection of the palatopharyngeus with the inferior constrictor may elucidate whether any anatomical structure directly acts on the UES opening.

This study aimed to examine the precise manner of the connection between the palatopharyngeus and inferior constrictor, as well as its histological characteristics. We hypothesized that the palatopharyngeus is interlaced with the cricopharyngeal part of the inferior constrictor, and its connection is histologically firm.

## Materials and methods

### Cadaver preparation

We evaluated 15 halves of 9 heads from Japanese cadavers (six men and three women; average age: 76.1 years) donated to our Department of Anatomy. The Ethics Committee at our institution approved the study design (D2018-055). All the cadaver specimens were fixed in 8% formalin and preserved in 30% ethanol. Of the 15 halves, 12 halves of 6 heads and 3 halves of 3 heads were randomly assigned for macroscopic and histological examinations, respectively. All cadavers used in this study had no history of craniofacial surgery or any other syndromes.

### Macroscopic examination

**Fig. 1:**
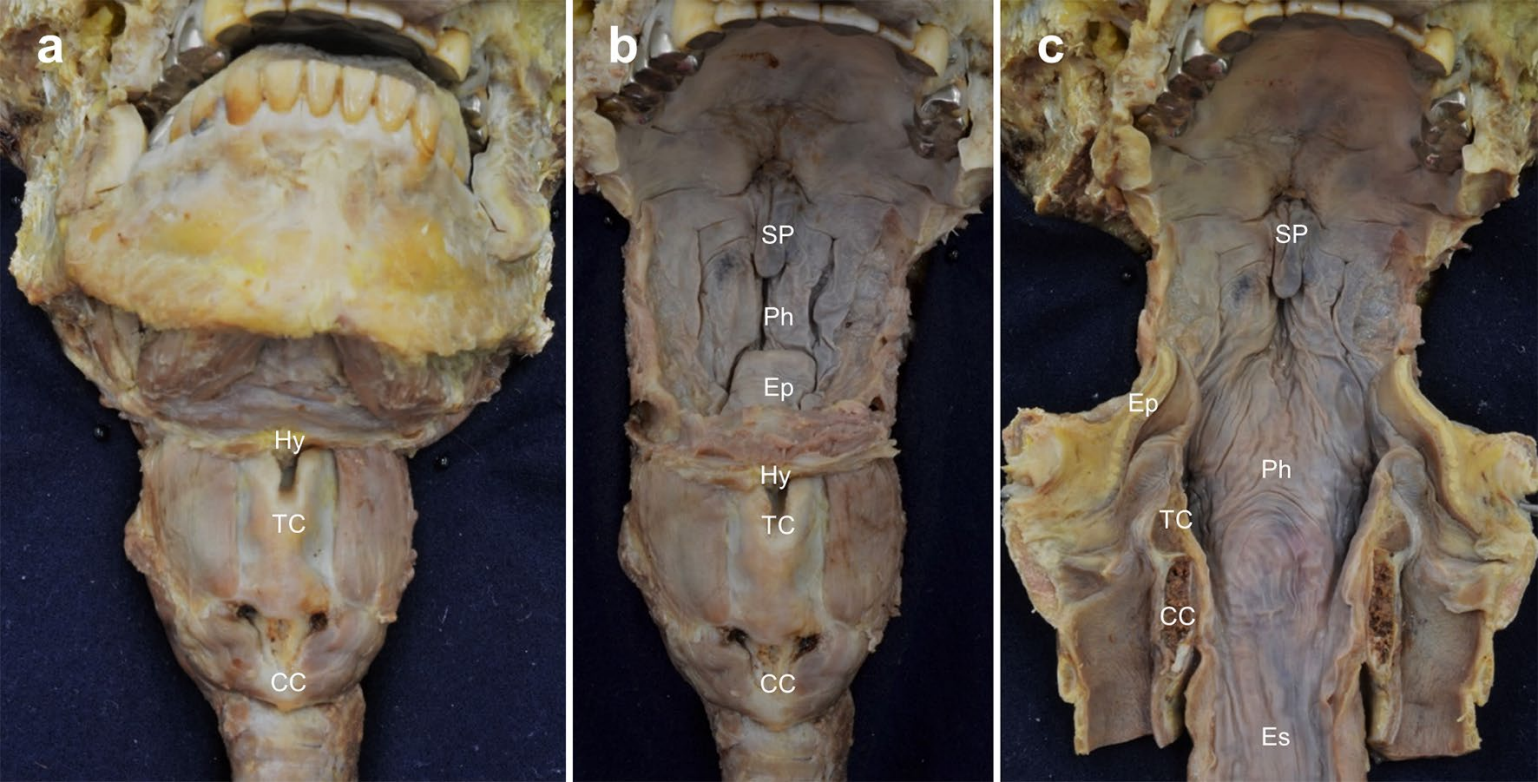
Dissection of the pharyngeal wall from its anterior aspect. **a** The anterior surface of the mandible, hyoid bone, thyroid cartilage, and cricoid cartilage is exposed. **b** Some parts of the mandible, tongue, and suprahyoid muscles are removed. **c** The hyoid bone, thyroid cartilage, cricoid cartilage, and anterior pharyngeal wall are cut in the midline and reflected laterally. *Hy* hyoid bone, *TC* thyroid cartilage, *CC* cricoid cartilage, *Ep* epiglottis, *Es* esophageal cavity, *Ph* pharyngeal cavity, *SP* soft palate

For macroscopic examination, the subcutaneous tissue, including the infrahyoid muscles, was removed for the visualization of the anterior surface of the mandible, hyoid bone, thyroid cartilage, and cricoid cartilage (Fig. 1a). Some parts of the mandible, tongue, and suprahyoid muscles were removed to observe the pharyngeal wall from the anterior view (Fig. 1b). The hyoid bone, thyroid, and cricoid cartilage were cut at the midline and reflected laterally to analyze the pharyngeal and esophageal walls (Fig. 1c). The pharyngeal and esophageal mucosa were removed for the visualization of the spatial arrangement of the palatopharyngeus.

### Histological examination

Two heads were used for histological analysis of the positional relationship between the palatopharyngeus and the inferior constrictor. We froze specimens at − 80 °C, and serially sectioned them into 5-mm thick segments in the sagittal plane using a band saw (WN-25-3; Nakajima Seisakusho, Osaka, Japan). We decalcified the blocks for 3 days in a solution containing aluminum chloride, hydrochloric acid, and formic acid, as described by Plank and Rychlo [21]. After dehydration, the blocks were embedded in paraffin and serially sectioned (5 μm thickness). The sections were stained with Elastica van Gieson to distinguish collagen from other connective tissues.

## Results

### Macroscopic examination

**Fig. 2:**
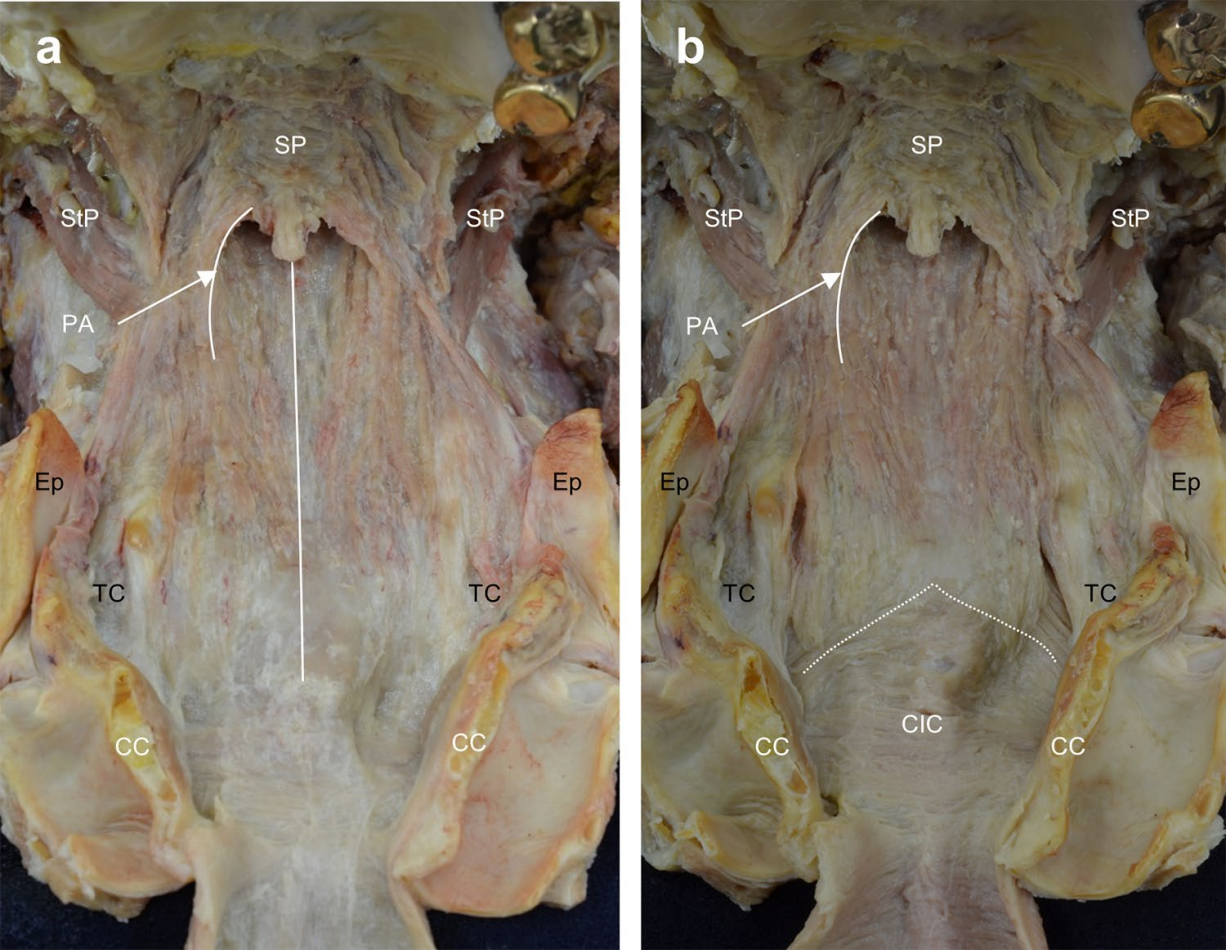
Spatial arrangement of the palatopharyngeus (anterior aspect). **a** The anterior aspect of the pharyngeal wall. The palatopharyngeus is partly spread on the inner surface of the inferior constrictor and connected to the fibrous tissue located at the level of the cricoid cartilage. **b** After removal of the fibrous tissue, the inferior end of the palatopharyngeus reveals extension into the inner surface of the cricopharyngeal part of the IC (CIC). *IC* inferior constrictor, Dotted line = border between the thyropharyngeal and cricopharyngeal parts of the IC, *Ep* epiglottis, Line = pharyngeal raphe, *PA* palatopharyngeal arch, *StP* stylopharyngeus, *SP* soft palate, *TC* thyroid cartilage, *CC* cricoid cartilage

The palatopharyngeus was found to originate from both the superior and inferior aspects of the palatine aponeurosis and formed the posterior and anterior parts of the palatopharyngeal arch, respectively. After forming the arch, the palatopharyngeus spread radially on the inner aspect of the pharyngeal wall, and, subsequently, inserted into the epiglottis, thyroid cartilage, and pharyngeal raphe. In addition, the most inferior portion of the palatopharyngeus descended inferomedially, and continued into the fibrous tissue located at the level of the cricoid cartilage (Fig. 2a). After the removal of the fibrous tissue, the inferior end of the palatopharyngeus was revealed. Its inferior end extended to the inner surface of the cricopharyngeal part of the inferior constrictor and covered its inner surface from the anterolateral end to the opposite inner surface of the anterolateral end (Fig. 2b).

### Histological examination

**Fig. 3:**
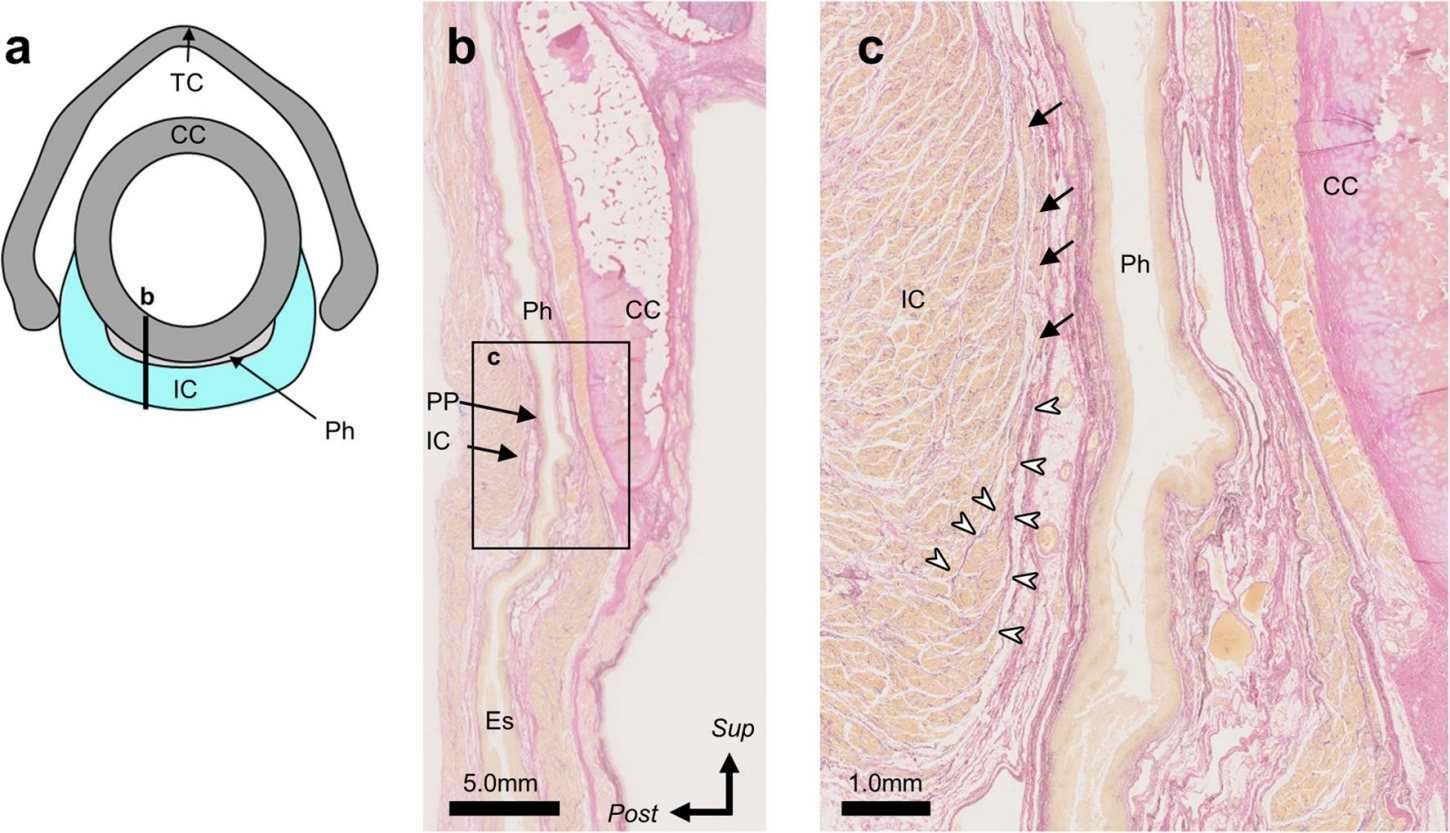
Histological analysis of the relationship between the palatopharyngeus and the inferior constrictor using Elastica van Gieson staining. **a** Schematic representation of the transverse section at the level of the cricoid cartilage. **b** The sagittal section along line b in 3a. **c** High magnification of the box c in 3b. The inferior end of the dense connective tissue of the palatopharyngeus, indicated by the white arrowhead, entered the muscle bundles of the inferior constrictor. Black arrow = muscle bundles of the palatopharyngeus, *CC* cricoid cartilage, *Es* esophageal cavity, *Ph* pharyngeal cavity, *PP* palatopharyngeus, *TC* thyroid cartilage, *IC* inferior constrictor, White arrowhead = dense connective tissue continuing from the inferior end of the palatopharyngeus*, Sup* Superior, *Post* Posterior

To evaluate the relationship between the palatopharyngeus and the inferior constrictor, sagittal histological sections were examined (Fig. 3a, b). At the level of the cricoid cartilage, the inferior end of the palatopharyngeus continued into the dense connective tissue, including the elastic fibers. These dense connective tissues not only covered the inner surface of the inferior constrictor but also entered into the muscle bundles and enveloped them (Fig. 3c).

## Discussion

The present study revealed that the most inferior portion of the palatopharyngeus extended into the inner surface of the cricopharyngeal part of the inferior constrictor. Histological analysis revealed that the dense connective tissue from the inferior end of the palatopharyngeus entered the muscle bundles of the inferior constrictor and wrapped around them at the level of the cricoid cartilage.

In general, the palatopharyngeus is inserted into the piriform fossa and the posterior border of the thyroid cartilage [3, 15–20]. Okuda et al. [17] have reported that the palatopharyngeus sometimes interlaces with the inferior constrictor. However, they did not report the precise manner of the connection. In this study, the most inferior portion of the palatopharyngeus extends into the inner surface of the cricopharyngeal part of the inferior constrictor, thereby indicating that the palatopharyngeus connects with the cricopharyngeal part of the inferior constrictor.

There have been some histological studies on the insertion of the palatopharyngeus. Meng et al. [16] have histologically shown that the palatopharyngeus is inserted into the piriform recess and the glossoepiglottic fold, together with the stylopharyngeus. However, there has been no histological analysis of the relationship between the palatopharyngeus and the inferior constrictor. In the present study, we clarified that the dense connective tissue, including elastic fibers, which continues from the inferior end of the palatopharyngeus, enters into the muscle bundles of the inferior constrictor and wraps them at the level of the cricoid cartilage. Hence, our histological findings suggest that the palatopharyngeus is closely connected to the inferior constrictor.

**Fig. 4:**
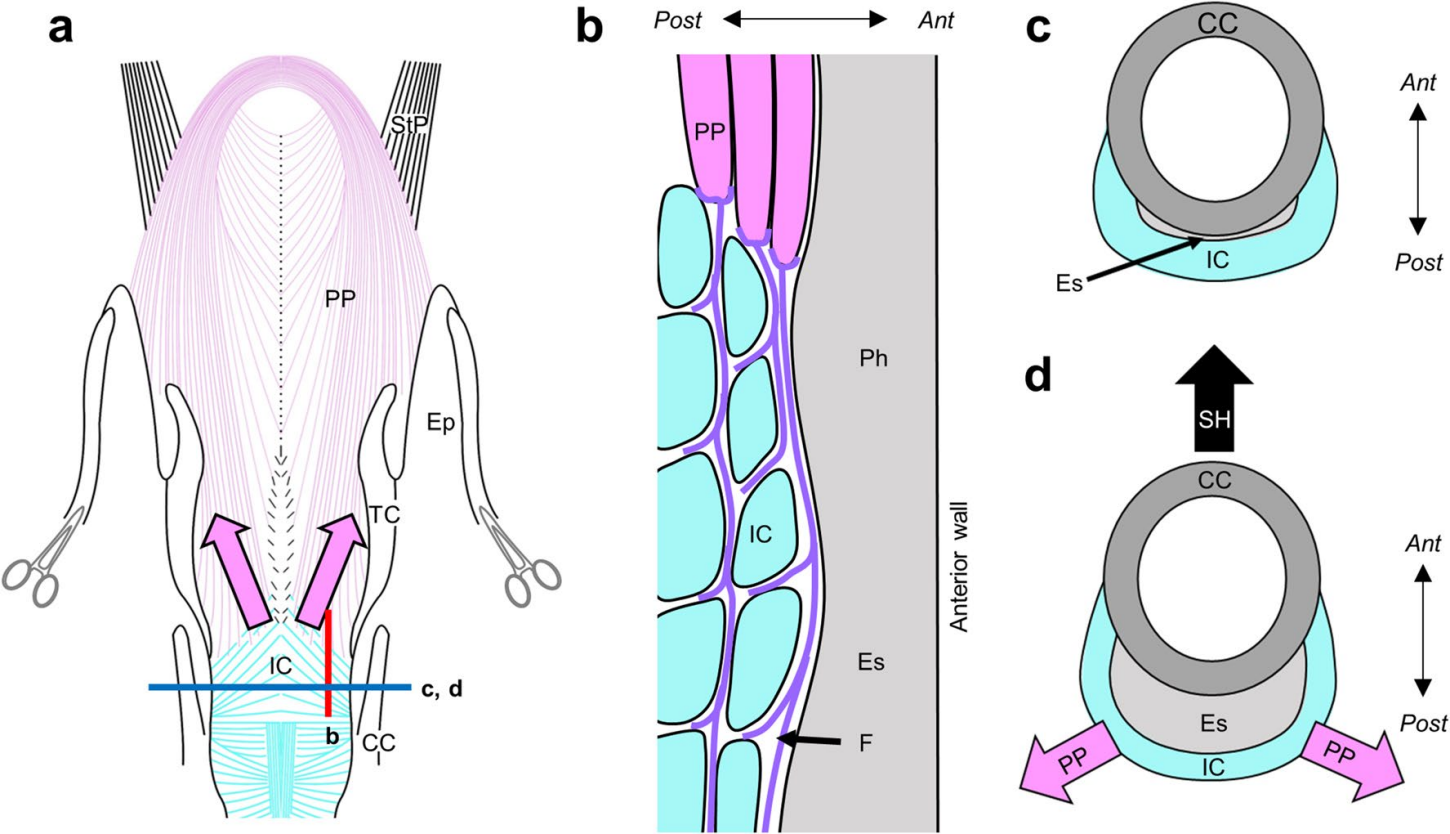
Schematic representation of the positional relationship between the palatopharyngeus and the inferior constrictor. **a** The anterior aspect of the pharyngeal wall. Pink arrows indicate the direction of the muscular force of the palatopharyngeus acting on the inferior constrictor. **b** A sagittal section of line b in 4a. **c** A transverse section of lines c and d in 4a during the closing of the UES. **d** A transverse section of lines c and d in 4a during the opening of the UES. Pink arrows indicate the direction of the muscular force of the palatopharyngeus acting on the inferior constrictor. The black arrow indicates the direction of the force of the suprahyoid muscles. *PP* palatopharyngeus, *IC* inferior constrictor*, Ep* epiglottis, *Es* Esophageal cavity, *F* fibrous connective tissue, *Ph* pharyngeal cavity, *SH* suprahyoid muscles, *StP* stylopharyngeus, *TC* thyroid cartilage, *Ant* Anterior, *Post* Posterior

The present study is clinically relevant, because it provides useful information on the UES opening during swallowing. In general, the UES opening has been considered as a passive event, because it occurs as a result of the elevation of the hyolaryngeal complex and of the relaxation of the cricopharyngeal part of the inferior constrictor [2, 7–11]. Our findings revealed that the palatopharyngeus spread across the inner surface of the cricopharyngeal part of the inferior constrictor, and it is connected to the cricopharyngeal part of the inferior constrictor thorough a dense connective tissue (Fig. 4a, b). These findings suggest that the bilateral palatopharyngeus might elevate the cricopharyngeal part of the inferior constrictor superolaterally, where the UES is generally located. With the aid of the pulling forces in the superoanterior direction by the suprahyoid muscles [22], the UES can be pulled efficiently in three different directions (Fig. 4c, d). Therefore, the palatopharyngeus might act directly on the UES opening in addition to the main mechanism, which results in a passive UES opening as a result of the elevation of the hyolaryngeal complex.

The present study has some limitations. First, it was a purely anatomical study and did not examine the muscle functions. Second, this study only used elderly cadavers. However, despite these limitations, we believe that our findings will contribute to a better understanding of the anatomical composition of the UES.

In conclusion, the most inferior portion of the palatopharyngeus extended to the inner surface of the cricopharyngeal part of the inferior constrictor. The dense connective tissue, which continues from the inferior end of the palatopharyngeus, entered into the muscle bundles of the inferior constrictor and wrapped them at the level of the cricoid cartilage. Therefore, the palatopharyngeus may also act on the UES opening directly and play an essential role in various events of swallowing.
